# Association of handgrip strength with the prevalence of metabolic syndrome in US adults: the national health and nutrition examination survey

**DOI:** 10.18632/aging.103097

**Published:** 2020-05-04

**Authors:** Chao Ji, Yang Xia, Shuhui Tong, Qijun Wu, Yuhong Zhao

**Affiliations:** 1 Department of Clinical Epidemiology, Shengjing Hospital of China Medical University, Shenyang, China; 2 Drilling and Production Technology Research Institute of Petrochina Liaohe Oilfield Company, Panjin, China

**Keywords:** metabolic syndrome, handgrip strength, waist circumference, triglyceride, hypertension

## Abstract

Objective: Aimed to find the cut-off point of handgrip strength and it’s association with MetS.

Results: The relative handgrip strength was negatively associated with the prevalence of MetS. Of note, the odds ratios (ORs) with 95% confidence intervals (CIs) across tertiles of relative handgrip strength were 1 (reference), 0.45 (0.33, 0.62), and 0.13 (0.08, 0.20) in male participants after adjusting for demographic factors, calorie intake, and physical activity. Similar results were observed in female participants. The cutoff values of relative handgrip strength for male and female participants were 0.52 and 0.40, respectively.

Conclusions: Findings of this study suggest that a strong relationship exists between handgrip strength and prevalence of MetS in US adults, regardless of sex.

Methods: A total of 5 056 participants in the National Health and Nutrition Examination Survey were analysed in this study. Handgrip strength was measured by using a handgrip dynamometer. MetS was defined in accordance with the criteria of the scientific statement of the American Heart Association in 2009. Multivariable binary logistic regression was used to explore the association between handgrip strength and MetS.

## INTRODUCTION

Metabolic syndrome (MetS) is a group of metabolic conditions that occur together and promote the development of cardiovascular disease and diabetes [1]. Moreover, MetS and its components are associated with cancers, such as colorectal cancer [2], prostate cancer [3], and primary liver cancer [4]. In parallel with changes towards a sedentary lifestyle and subsequent obesity worldwide, the prevalence of MetS has increased in recent decades. A previous study demonstrated that the prevalence of MetS in the United States (US) has increased from 32.9% in 2003-2004 to 34.7% in 2010-2012 [5]. Thus, considering the increasing burden of MetS, it is important to identify modifiable risk factors of MetS.

Handgrip strength is a simple and reliable measurement method, especially a good measurement of upper body strength, which is realized as an index for muscular fitness [6, 7]. And a strong evidence suggests that in addition to obesity, low level of fitness is a major contributor to a development of metabolic disease and metabolic syndrome [8, 9]. Furthermore, muscle strengthening activities are closely related with improved insulin sensitivity, meliorated dyslipidemia, and reduced blood pressure, all of those are principle components of metabolic syndrome [10–12]. In addition, skeletal muscle can secrete multiple peptides [13]. Many of these peptides contribute to metabolic homeostasis [14, 15]. Interleukin-15 from skeletal muscle negatively regulates fat mass [16]. Inhibition of myostatin suppresses body fat accumulation and improves insulin sensitivity [17, 18]. These myokines appear to participated in maintenance of whole-body metabolic homeostasis. Muscles are target organs for insulin action [19]. Moreover, insulin resistance is a hallmark of MetS [20]. Muscles also secrete irisin [21] which could be a therapeutic tool in managing obesity and MetS [22]. Thus, it is plausible that muscle strength could play an important role in the development of MetS. Indeed, several previous studies found that high muscle strength was associated with lower prevalence of MetS [23–30]. Most of these studies were conducted in Asian countries. In the US, only the Aerobics Center Longitudinal Study demonstrated that lower muscle strength was associated with higher MetS prevalence in men [30]. Moreover, compared to participants with higher muscle strength, the odds ratios (ORs) were 2.20 (95% confidence interval (CI), 1.89-2.54) and 2.11 (95% CI, 1.62-2.74) for male participants aged <50 years and ≥50 years with lower muscle strength, respectively [30]. However, the Aerobics Center Longitudinal Study used the supine bench press and seated leg press to assess muscle strength [30] instead of handgrip strength, which is easier to use in both clinical and community settings [31]. Moreover, most of the previous studies were conducted in middle-aged and older participants or in men alone, which would limit the generalization of their findings. Studies have shown that BMI increased with the prevalence of MetS [32]. BMI can be a factor differentiating the course of many metabolic and adaptive processes in the body [33–36]. It can also be a factor conditioning a different response of the body to certain lifestyle elements that modifiers of metabolic risk factors [37]. We therefore accept the hypothesis that individuals with a different BMI also have a different response to the relationship between muscle strength and MetS.

To the best of our knowledge, no epidemiological study has investigated the associations between handgrip strength and MetS in general US adults and considering BMI as a stratification factor. Thus, we used data from 2011-2012 and 2013-2014 survey cycles of the National Health and Nutrition Examination Survey (NHANES) to determine the associations between handgrip strength and MetS and identify cut-off points of handgrip strength associated with MetS in general US adults. In order to help individuals to identify the risk of MetS as quick as possible.

## RESULTS

### Characteristics of participants

According to the data from NHANES 2011-2012 and 2013-2014 cycles, 19 931 people joined the household questionnaire interview, and 14 984 people participated in the handgrip strength test. Because of the design of NHANES, subsampling was required to reduce respondent burden and facilitate the scheduling and completion of examinations, the final analysed sample comprised 5 056 participants, of which 36.33% had MetS. The prevalence of MetS in men was 36.35% and that in women was 36.30% (*P*=0.9796). Baseline characteristics of the participants are shown in Table 1. With the increase in relative handgrip strength, age, BMI, waist circumference, triglyceride, fasting glucose, SBP, and the prevalence of MetS were decreased, and the proportion of male participants, DBP, tobacco and alcohol use, proportion of college graduation, percentage of >$100 000 annual household income, energy intake per day, and proportion of high physical activity were increased.

**Table 1:** Participants characteristics according to categories of adjusted handgrip.

	**Tertiles of handgrip strength per weight (kg/kg) (Range)**					*P* for trend*
	**Level 1 (0.11, 0.41)**		**Level 2 (0.41, 0.53)**		**Level 3 (0.53, 1.13)**	
No. of participants (%)	33.32		33.56		33.12	
Age (year)	49.92 (49.01, 50.84)		41.47 (40.56, 42.38)		35.29 (34.38, 36.20)	<0.0001
Male (%)	21.31		47.69		80.69	<0.0001
BMI (kg/m^2^)	33.41 (33.15, 33.67)		26.51 (26.24, 26.77)		23.79 (23.53, 24.06)	<0.0001
Waist circumference (cm)	108.28(107.60, 108.96)		93.00 (92.32, 93.68)		85.16 (84.48, 85.84)	<0.0001
Triglyceride (mg/dL)	125.16 (120.18, 130.13)		116.84 (111.870, 121.82)		104.59 (99.63, 109.56)	<0.001
HDL cholesterol (mg/dL)	52.46 (51.76, 53.15)		53.76 (53.07. 54.46)		53.62 (52.93 54.32)	0.0204
Fasting glucose (mg/dL)	112.39 (110.98, 113.81)		102.80 (101.38, 104.22)		99.40 (97.99, 100.82)	<0.0001
SBP (mm Hg)	123.43 (122.64, 124.22)		118.79 (118.00, 119.59)		117.28 (116.49,118.07)	<0.0001
DBP (mm Hg)	67.32 (66.70, 67.94)		67.07 (66.45, 67.69)		67.18 (66.57, 67.80)	0.7609
Metabolic syndrome (%)	59.35		33.48		16.06	<0.0001
Smoke						
Every day (%)	34.21		31.63		47.26	<0.0001
Somedays (%)	6.12		8.83		8.81	
Never (%)	59.67		59.54		43.93	
Drink						
Yes (%)	70.37		80.97		85.55	<0.0001
No (%)	29.63		19.03		14.45	
Education						
≤High school (%)	43.95		42.74		46.80	0.0971
≥College (%)	56.05		52.26		53.20	
Annual household income (%)						
Low (<35 000)	40.56		29.91		30.45	<0.0001
Medium (<100 000)	44.25		43.74		42.23	
High (≥100 000)	15.19		26.34		27.32	
Energy intake (kcal/d)	1899.01 (1845.31, 1952.71)		2120.94 (2067.18, 2174.71)		2388.95 (2335.31, 2442.59)	<0.0001
Physical activity (MET * h/wk) (%)						
Low (< 3500/year)	47.65		29.44		19.95	<0.0001
Medium (< 10 000/year)	31.07		34.28		34.14	
High (≥ 10 000/year)	21.28		36.28		45.91	

BMI, body mass index; DBP, diastolic blood pressure; MET, metabolic equivalent; SBP, systolic blood pressure.

* *P* for trend was anayzed by variance or chis-quare test.

### Handgrip strength and MetS

The association between relative handgrip strength and components of MetS in the male and female participants is shown in Table 2. Multivariable binary logistic regression was used to estimate the OR value and 95% CI. Relative handgrip strength was negatively associated with the prevalence of MetS in male and female participants, the OR (95% CI) values across the tertiles of relative handgrip strength were 1 (Reference), 0.45 (0.33, 0.62), 0.13 (0.08, 0.20) for male participants (*P*_for trend_ <0.0001) and 1 (Reference), 0.35 (0.27, 0.46), 0.12 (0.08, 0.18) for female participants (*P*_for trend_ <0.0001) after adjusting for age, race, drinking and smoking status, education level, household income, total energy intake, and physical activity. We additionally analysed the association between handgrip strength and components of MetS, and the results were comparable in both male and female participants.

**Table 2:** Association between handgrip and metabolic syndrome and its components in men and women.

	**Men (N=2535)**						**Women (N=2521)**					
	**Tertiles of handgrip strength per body weight (kg/ kg) (range)**					**P for trend^c^**		**Tertiles of handgrip strength per body weight (kg/ kg) (range)**					**P for trend^c^**
	**Level 1 (0.13, 0.48)**		**Level 2 (0.48, 0.60)**		**Level 3 (0.60, 1.13)**			**level 1 (0.11, 0.36)**		**Level 2 (0.36, 0.46)**		**Level 3 (0.46, 0.77)**	
No. of participants (%)	31.80		34.33		33.86			33.44		34.73		31.83	
No. of participants with metabolic syndrome (%)	61.50		37.24		11.84	<0.0001		65.11		32.60		10.08	<0.0001
Crude model	Reference		0.37 (0.28, 0.50) ^b^		0.08 (0.06, 0.12)	<0.0001		Reference		0.26 (0.21, 0.32)		0.06 (0.04, 0.09)	<0.0001
Adjusted model ^a^	Reference		0.45 (0.33, 0.62)		0.13 (0.08, 0.20)	<0.0001		Reference		0.35 (0.27, 0.46)		0.12 (0.08, 0.18)	<0.0001
No. of participants with elevated waist circumference (%)	75.88		40.28		6.67	<0.0001		95.28		71.05		22.00	<0.0001
Crude model	Reference		0.21 (0.16, 0.28)		0.02 (0.02, 0.03)	<0.0001		Reference		0.12 (0.08, 0.20)		0.01 (0.01, 0.02)	<0.0001
Adjusted model ^a^	Reference		0.22 (0.17, 0.29)		0.03 (0.02, 0.03)	<0.0001		Reference		0.14 (0.09, 0.23)		0.02 (0.01, 0.03)	<0.0001
No. of participants with elevated triglycerides (%)	56.85		40.68		22.90	<0.0001		53.63		29.76		16.44	<0.0001
Crude model	Reference		0.52 (0.39, 0.70)		0.23 (0.17, 0.30)	<0.0001		Reference		0.37 (0.29, 0.47)		0.17 (0.12, 0.24)	<0.0001
Adjusted model ^a^	Reference		0.67 (0.46, 0.98)		0.39 (0.28, 0.53)	<0.0001		Reference		0.52 (0.40, 0.69)		0.40 (0.28, 0.58)	<0.0001
No. of participants with lower HDL (%)	29.14		25.21		17.47	<0.0001		42.05		30.27		19.38	<0.0001
Crude model	Reference		0.82 (0.63, 1.07)		0.51 (0.36, 0.73)	0.0004		Reference		0.60 (0.48, 0.74)		0.33 (0.26, 0.43)	<0.0001
Adjusted model ^a^	Reference		0.81 (0.61, 1.08)		0.51 (0.34, 0.76)	0.0013		Reference		0.49 (0.38, 0.6)		0.22 (0.15, 0.33)	<0.0001
No. of participants with elevated blood pressure (%)	56.77		40.05		22.38	<0.0001		58.03		31.49		14.68	<0.0001
Crude model	Reference		0.51 (0.37, 0.70)		0.22 (0.17, 0.28)	<0.0001		Reference		0.33 (0.27, 0.41)		0.13 (0.10, 0.16)	<0.0001
Adjusted model ^a^	Reference		0.70 (0.49, 0.99)		0.37 (0.28, 0.50)	<0.0001		Reference		0.49 (0.34, 0.69)		0.33 (0.23, 0.48)	<0.0001
No. of participants with elevated fasting glucose (%)	65.16		51.63		33.98	<0.0001		56.09		34.56		17.55	<0.0001
Crude model	Reference		0.57 (0.44, 0.74)		0.28 (0.22, 0.35)	<0.0001		Reference		0.41 (0.32, 0.53)		0.17 (0.13, 0.22)	<0.0001
Adjusted model ^a^	Reference		0.71 (0.52, 0.96)		0.44 (0.32, 0.61)	<0.0001		Reference		0.56 (0.42, 0.75)		0.33 (0.25, 0.45)	<0.0001

a. Logistic regression adjusted for age, race, drinking status, smoking status, education level, income, total energy intake, and physical activity.

b. Odds ratio (95% confidence interval) (all such values).

HDL, high-density lipoprotein.

c. *P* for trend was analysed by multiple logistic regression analysis.

To analyse the effect of BMI on the association between handgrip strength and components of MetS, we categorized BMI to lower BMI (BMI < 30) and higher BMI (BMI ≥ 30) groups. Supplementary Table 1 shows the results of the association between relative handgrip strength and components of MetS in male participants. Handgrip strength was negatively associated with the prevalence of MetS in male participants with BMI < 30 after adjusted by potential covariables. However, in male participants with BMI ≥ 30, the negative association was lost. In female participants, handgrip strength was negatively associated with the prevalence of MetS, in both BMI < 30 group and BMI ≥ 30 group (Supplementary Table 2).

**Figure 1:**
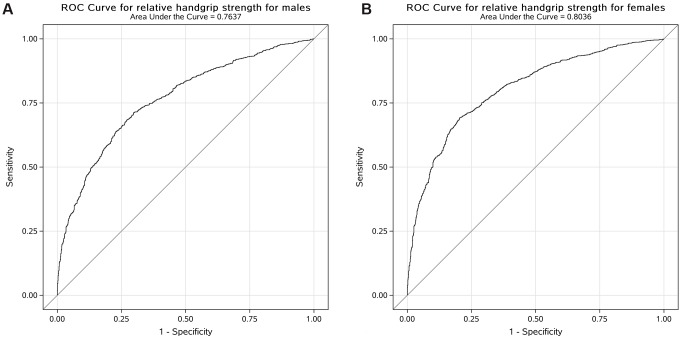
Receiver operating characteristic curve for the adjusted model to analyse the association between relative handgrip strength and prevalence of MetS in US male (**A**) and female (**B**) participants.

The ROC curve was used to evaluate the performance of the adjusted model to analyse the association between relative handgrip strength and prevalence of MetS. The AUC with 95% CI for each adjusted model in both male and female participants is shown in Figure 1. The AUC value of the adjusted model for male participants was 0.76 (95% CI: 0.74, 0.78) and that for female participants was 0.80 (95% CI: 0.79, 0.82). The cut-off values of relative handgrip strength were 0.52 and 0.40 for male and female participants, respectively.

## DISCUSSION

In this cross-sectional study, we explored the associations between handgrip strength and MetS in general US adults. The results demonstrated that higher handgrip strength was associated with lower prevalence of MetS in both male and female participants after adjustments of age, race, drinking status, smoking status, educational level, household income, total energy intake, and physical activity. We further generated ROC curves to quantify the AUC and the cut-off points of handgrip strength associated with the prevalence of MetS in both male and female participants.

In this study, the prevalence rates of MetS were 36.4% and 36.3% in US men and women, respectively. The results were close to the last report using data from the 2011-2012 wave of the NHANES, which found that the prevalence rates of MetS in the US were 32.8% and 36.6% in men and women, respectively [5].

Handgrip strength is a simple and reliable measurement of upper body strength and muscle mass [29]. Moreover, compared to supine bench press and seated leg press, handgrip strength is much easier to assess. Thus, handgrip strength could be a flexible indicator for screening MetS. A few previous studies have investigated the associations between handgrip strength and prevalence of MetS in different populations [24, 25, 27, 29]. A cross-sectional study conducted in Australian men found that the odds for the prevalence of MetS increased for lower muscle strength per body weight [24]. Another study conducted in Korea found that grip strength per body weight was lower in participants with MetS than in those without MetS [25]. A large Chinese population-based study also found that handgrip strength per body weight was inversely associated with MetS [27]. The ORs (95% CI) of MetS across decreasing handgrip strength per weight quartiles were 1.00 (reference), 1.87 (1.66, 2.11), 2.40 (2.13, 2.71), and 3.36 (2.97, 3.80) in men and 1.00 (reference), 1.80 (1.48, 2.21), 2.77 (2.29, 3.36), and 3.89 (3.22, 4.71) in women [27]. Most recently, another cross-sectional study including 488 male and 521 female participants found that low handgrip strength per body weight was associated with higher prevalence of MetS [29]. Compared with participants in the highest tertile of handgrip strength per weight, those in the lowest tertile had 2.52 (95% CI, 1.43-4.46) and 5.01 (95% CI, 1.66-15.08) times higher prevalence of MetS in male and female participants, respectively [29].

In line with previous studies, we found that higher handgrip strength per body weight was associated with lower prevalence of MetS after adjustments of confounding factors in the present study. The mechanisms that have been proposed to explain this association are mainly related to insulin resistance and inflammation. Muscles are major sites of insulin-stimulated whole-body glucose disposal and muscle metabolism can influence whole-body glucose homeostasis and insulin sensitivity [38]. Meanwhile, insulin resistance is the most accepted unifying theory explaining the pathophysiology of MetS [39]. Moreover, low muscle strength and mass were associated with high level of inflammation markers, such as C-reactive protein [40] and neutrophil-lymphocyte ratio [41]. A chronic state of inflammation also appears to be a central mechanism underlying the pathophysiology of insulin resistance and MetS [42]. Previous studies also demonstrated that skeletal muscles could release factors such as irisin [21] and myonectin [43], which can exert endocrine effects and consequently contribute to risk factors of MetS [44, 45]. Thus, lower muscle strength and mass could lead to the development of MetS.

Furthermore, the ROC curves were generated to determine the cut-off points of handgrip strength per body weight associated with MetS. Results suggested that the cut-off points were 0.52 and 0.40 kg/kg of handgrip strength per body weight in men and women, respectively. The results were in line with a previous study that analysed 17,703 Chinese adults and found that the optimal cut-off values were 0.56 and 0.40 in men and women, respectively [27]. Another study found that the cut-off points were 2.57 and 2.35 kg/kg bench press and leg press per body weight in US men aged < 50 years and ≥ 50 years [30]. However, to the best of our knowledge, this is the first study that used handgrip strength, which is easier to employ in both clinical and community settings, to calculate cut-off points of muscle strength associated with MetS.

Some limitations are notable in this study. Firstly, given the nature of observational study design, the mechanism underlying the associations cannot be illuminated. Secondly, even though we have adjusted confounding factors, we cannot rule out the possibility that unmeasured factors might contribute to the associations observed. Thirdly, selection bias may exist because lots of participates were excluded from the study. However, NHANES is unique in collecting person-level demographic, health, and nutrition information from personal interviews (interview) and a standardized physical examination in a mobile examination center (MEC). The examination includes objective measures of health status, including height, weight, blood pressure, and the collection of blood and urine specimens for laboratory testing. For some of these components, subsampling was required to reduce respondent burden and facilitate the scheduling and completion of examinations. In addition, weights were assigned to each component during sampling and analysis process. So sample representativeness was not been affected. Furthermore, considering the different definitions of MetS between studies, the comparability and generality of this study was limited. Finally, this study was conducted in a population of US adults, which could limit the generalizability of results to other populations.

In conclusion, findings from this study show that relative handgrip is negatively associated with the prevalence of MetS which defined by a joint multinational interim statement in men with BMI < 30 and women. Handgrip strength per weight lower than 0.52 (kg/ kg) in men and 0.40 (kg/ kg) in women may increase the risk of MetS. Enhancing muscle strength activities may be a new perspective for prevent MetS. Handgrip strength test may be a simple way to identify MetS. And further random controlled trial studies and cohort studies were needed.

## MATERIALS AND METHODS

### Study population

NHANES, a program of studies conducted by the Centers for Disease Control and Prevention’s (CDC) National Center for Health Statistics (NCHS), was designed to assess the health and nutritional status of adults and children in the US. NHANES uses a complex, multistage, probability sampling design to select participants representative of the civilian, non-institutionalized US population. Household interviews and physical and laboratory examinations were included in the survey [46]. Handgrip strength data are available for 2011-2012 and 2013-2014 survey cycles. Participants aged ≥20 years were included. All adults provided written consent to participate in NHANES, which was reviewed and approved by the NCHS Research Ethics Review Board.

### Handgrip strength

Handgrip strength was measured using a Takei Digital Grip Strength Dynamometer (T.K.K. 5401) to the nearest 0.1 kg. Participants were asked to remove hand and wrist jewellery and adjust the grip size of the dynamometer until the second joint of the index finger was at 90°. After a practice trial, each hand of the participants would be tested three times, alternating hands between trials with a 60-s rest between measurements on the same hand. Participants stood straight with their feet and hip apart and arms fully extended alongside with palms facing their thighs. The participant’s hand, which was in line with the wrist and forearm, quickly and forcefully squeezed the dynamometer handle at their maximum strength. The complete handgrip strength testing protocol is described in the NHANES muscle strength procedure manual (https://wwwn.cdc.gov/nchs/nhanes). To avoid the potential bias effect of body weight on the estimation of handgrip strength, relative handgrip strength (handgrip/body weight) was calculated to assess the handgrip strength.

### Metabolic syndrome

MetS was defined according to the joint scientific statement of harmonizing the MetS criteria as participants presented three or more of the following components [47]: (1) waist circumference ≥88 cm in women and ≥102 cm in men; (2) elevated plasma triglycerides ≥150 mg/dl (1.7 mmol/L), or treatment for elevated triglycerides; (3) reduced high-density lipoprotein cholesterol <40 mg/dl (1.0 mmol/L) in men, and <50 mg/dl (1.3 mmol/L) in women, or treatment for reduced high-density lipoprotein cholesterol; (4) evaluated blood pressure (BP): systolic blood pressure (SBP) ≥130 mm Hg and/or diastolic blood pressure (DBP) ≥85 mm Hg, or antihypertensive drug treatment; (5) high fasting glucose ≥100 mg/dl, or drug treatment for elevated glucose.

### Assessment and definition of other variables

Age, gender, race, education, household income, diet, physical activity, and tobacco and alcohol use were self-reported. Race was classified as Mexican American, other Hispanic, Non-Hispanic White, Non-Hispanic Black, and other Race. Education level was categorized into high school and lower (≤3) and college graduate and above (4-5). Household income was categorized as low (< 3500/year), medium (< 100000/year), and high (≥ 100000/year).

Standing height was measured to the nearest 0.1 cm by a stadiometer platform. A digital weighing scale was used to measure weight to the nearest 0.1 kg. Body mass index (BMI) was calculated as weight in kilograms divided by height in meters squared. Waist circumference was measured at the horizontal plane of the iliac crest to the nearest millimetre, using a measuring tape [48].

Seated resting BP was obtained by a trained physician using a Baumanometer^®^ calibrated mercury true gravity wall model sphygmomanometer and Baumanometer^®^ Calibrated V-Lok® cuffs. All participants sat all the way to the back of the height-adjustable chair to keep the spine straight and rested quietly for 5 min prior to BP measurement. The arm was bare and unrestricted by clothing with the palm turned upward. The elbow was slightly flexed, and the midpoint of the upper arm was at the level of the heart. Three consecutive BP readings were obtained by auscultation. A fourth attempt was performed if a BP measurement was interrupted or incomplete. All BP determinations (systolic and diastolic) were performed in the NHAES Mobile Examination Center.

Physical activity was assessed using the Global Physical Activity Questionnaire. Participants were asked to report the frequency and duration of leisure-time physical activities in a typical week. According to physical activity guidelines [49], vigorous-intensity activity and moderate-intensity activity were translated to metabolic equivalent values; therefore, the total physical activity (metabolic equivalent value per week) for each participant was calculated. Dietary intake was assessed via two 24-h recall interviews. Energy intake was later quantified for each food recorded during the survey.

### Statistical analysis

Participants’ baseline characteristics have been presented as least square means (with 95% confidence interval, CI) for continuous variables. Categorical variables were presented as percentages. Relative handgrip strength was categorized into sex-specific tertiles and sex-BMI-specific tertiles in separate models. Multivariable logistic regression was used to estimate the ORs with 95% CIs of associations between tertiles of relative handgrip strength and MetS with each component. The linear trend across increasing tertiles was tested using the median value of each tertile as a continuous variable based on logistic regression. The crude model was unadjusted. To estimate the potential differences in confounding effects, we adjusted for covariates including age, race, drinking and smoking status, education level, household income, total energy intake, and physical activity. The area under receiver operating characteristic (ROC) curve (AUC) with 95% CI was calculated to assess the performance of the multivariable logistic model. The cutoff value of relative handgrip strength associated with MetS was quantified by ROC curve.

All models were estimated using appropriate SAS survey procedures and NHANES strata, cluster, and sampling probability weights to account for the NHANES complex survey design and to produce unbiased national estimates. Sampling probability weights were appropriately constructed based on NHANES guidance. All data management and analyses were performed using SAS version 9.4 (SAS Institute Inc., Cary, NC). Two-sided P<0.05 was considered significant.

### Research data (data sharing and collaboration)

NHANES is an open database. All the data from this study could be acquired through the website https://www.cdc.gov/nchs/nhanes/.

## Supplementary Material

Supplementary Tables
