# Supplementary material for: Association of handgrip strength with the prevalence of metabolic syndrome in US adults: the national health and nutrition examination survey
Source: Aging (Albany NY). 2020 May 4;12(9):7818–29. doi: 10.18632/aging.103097 (PMC7244054; doi:10.18632/aging.103097)
Supplement: Supplementary Tables [file aging-12-103097-s001..pdf]

SUPPLEMENTARY TABLES

Supplementary Table 1. The association between handgrip and metabolic syndrome and its components in men.

	Men (BMI<30)				Men (>=30)			
	Tertiles of handgrip strength per body weight (kg/kg) (range)			P for trend [c]	Tertiles of handgrip strength per body weight (kg/kg) (range)			P for trend [c]
	Level1 (0.20, 0.53)	Level2 (0.53, 0.64)	Level3 (0.64, 1.13)		Level1 (0.14, 0.39)	Level2 (0.40, 0.48)	Level3 (0.48, 0.87)	
No. of participants (%)	30.85	34.89	34.26		29.48	34.04	36.48	
No. of participants with metabolic syndrome (%)	38.74	20.71	7.00	<0.0001	74.99	72.73	61.56	0.0043
Crude model	Reference	0.41 (0.27, 0.63) [b]	0.12 (0.08, 0.18)	<0.0001	Reference	0.89 (0.52, 1.54)	0.53 (0.31, 0.92)	0.0209
Adjusted model [a]	Reference	0.59 (0.35, 0.98)	0.24 (0.14, 0.41)	<0.0001	Reference	0.90 (0.49, 1.66)	0.89 (0.51, 1.57)	0.7150
No. of participants with elevated waist circumference (%)	34.76	16.94	1.81	<0.0001	98.48	98.10	79.56	<0.0001
Crude model	Reference	0.38 (0.26, 0.57)	0.04 (0.01, 0.09)	<0.0001	Reference	0.80 (0.26, 2.41)	0.06 (0.02, 018)	<0.0001
Adjusted model [a]	Reference	0.47 (0.32, 0.70)	0.06 (0.02, 0.15)	<0.0001	Reference	0.52 (0.19, 1.45)	0.05 (0.02, 0.11)	<0.0001
No. of participants with elevated triglycerides (%)	45.21	32.56	18.55	<0.0001	66.33	58.35	50.86	0.0049
Crude model	Reference	0.58 (0.40, 0.84)	0.28 (0.18, 0.44)	<0.0001	Reference	0.74 (0.44, 1.15)	0.53 (0.29, 0.96)	0.0382
Adjusted model [a]	Reference	0.80 (0.50, 1.30)	0.52 (0.29, 0.92)	0.0239	Reference	0.70 (0.44, 1.09)	0.83 (0.44, 1.57)	0.6532
No. of participants with lower HDL (%)	23.81	17.59	15.20	<0.0001	39.76	29.67	36.96	0.3933
Crude model	Reference	0.68 (0.46, 1.00)	0.58 (0.36, 0.92)	0.0209	Reference	0.64 (0.35, 1.15)	0.89 (0.52, 1.53)	0.7373
Adjusted model [a]	Reference	0.76 (0.52, 1.10)	0.67 (0.38, 1.16)	0.1376	Reference	0.66 (0.33, 1.31)	0.79 (0.42, 1.49)	0.5198
No. of participants with elevated blood pressure (%)	45.21	30.41	20.60	<0.0001	62.75	62.24	46.45	0.0007
Crude model	Reference	0.53 (0.37, 0.75)	0.32 (0.24, 0.42)	<0.0001	Reference	0.98 (0.63, 1.54)	0.51 (0.33, 0.81)	0.0056
Adjusted model [a]	Reference	0.79 (0.50, 1.24)	0.64 (0.41, 0.99)	0.0388	Reference	1.16 (0.64, 2.10)	0.96 (0.53, 1.75)	0.8161
No. of participants with elevated fasting glucose (%)	53.77	43.18	29.80	<0.0001	74.77	68.64	61.81	0.0117
Crude model	Reference	0.66 (0.46, 0.95)	0.36 (0.27, 0.48)	<0.0001	Reference	0.74 (0.40, 1.36)	0.55 (0.33, 0.91)	0.0225
Adjusted model [a]	Reference	0.88 (0.58, 1.35)	0.58 (0.42, 0.81)	0.0026	Reference	0.94 (0.52, 1.70)	0.0.97 (0.55, 1.73)	0.9568

a. Logistic regression adjusted for age, race, drinking status, smoking status, education level, income, total energy intake and physical activity.
b. Odds ratio (95% confidence interval) (all such values).
BMI, body mass index.
c. *P* for trend was analysed by multiple logistic regression analysis.

Supplementary Table 2. The association between handgrip and metabolic syndrome and its components in women.

	Women (BMI<30)				Women (BMI>=30)			
	Tertiles of handgrip strength per body weight (kg/ kg) (range)			P for trend [c]	Tertiles of handgrip strength per body weight (kg/ kg) (range)			P for trend [c]
	Level1 (0.14, 0.42)	Level2 (0.42, 0.50)	Level3 (0.50, 0.77)		Level1 (0.11, 0.29)	Level2 (0.29, 0.35)	Level3 (0.35, 0.69)	
No. of participants (%)	35.66	32.60	31.74		32.70	34.04	33.26	
No. of participants with metabolic syndrome (%)	41.10	16.41	6.73	<0.0001	71.87	64.21	48.55	<0.0001
Crude model	Reference	0.28 (0.18, 0.45) [d]	0.10 (0.06, 0.18)	<0.0001	Reference	0.70 (0.47, 1.06)	0.37 (0.26, 0.53)	<0.0001
Adjusted model [a]	Reference	0.59 (0.36, 0.98)	0.31 (0.16, 0.60)	0.0003	Reference	0.84 (0.54, 1.31)	0.65 (0.44, 0.98)	0.0402
No. of participants with elevated waist circumference (%)	71.74	41.30	12.83	<0.0001	100.00	99.92	99.73	0.1573
Crude model	Reference	0.28 (0.21, 0.36)	0.06 (0.04, 0.09)	<0.0001	Reference	-	-	-
Adjusted model [a]	Reference	0.36 (0.27, 0.50)	0.09 (0.05, 0.14)	<0.0001	Reference	-	-	-
No. of participants with elevated triglycerides (%)	43.25	21.15	12.99	<0.0001	56.94	51.19	30.13	<0.0001
Crude model	Reference	0.57 (0.32, 1.02)	0.38 (0.22, 0.66)	<0.0001	Reference	0.98 (0.58, 1.65)	0.53 (0.30, 0.92)	<0.0001
Adjusted model [a]	Reference	0.73 (0.47, 1.13)	0.59 (0.35, 1.00)	0.0184	Reference	0.97 (0.70, 1.33)	0.56 (0.36, 0.85)	0.0087
No. of participants with lower HDL (%)	23.74	22.76	18.45	0.0039	51.30	45.23	41.19	0.1446
Crude model	Reference	0.95 (0.64, 1.39)	0.73 (0.53, 0.99)	0.0502	Reference	0.78 (0.50, 1.23)	0.72 (0.50, 1.04)	0.0833
Adjusted model [a]	Reference	0.80 (0.53, 1.20)	0.59 (0.38, 0.91)	0.0170	Reference	0.67 (0.41, 1.12)	0.55 (0.35, 0.85)	0.0087
No. of participants with elevated blood pressure (%)	42.79	21.20	12.79	<0.0001	63.94	48.36	38.73	<0.0001
Crude model	Reference	0.36 (0.26, 0.50)	0.20 (0.15, 0.26)	<0.0001	Reference	0.53 (0.34, 0.82)	0.36 (0.23, 0.55)	<0.0001
Adjusted model [a]	Reference	1.00 (0.59, 1.70)	0.98 (0.56, 1.73)	0.9459	Reference	0.56 (0.32, 0.97)	0.56 (0.32, 0.98)	0.0465
No. of participants with elevated fasting glucose (%)	39.23	24.19	13.88	<0.0001	62.77	51.98	47.12	<0.0001
Crude model	Reference	0.50 (0.35, 0.70)	0.25 (0.19, 0.33)	<0.0001	Reference	0.64 (0.42, 0.98)	0.53 (0.34, 0.81)	0.0058
Adjusted model [a]	Reference	0.92 (0.59, 1.43)	0575 (0.38, 0.85)	0.0186	Reference	0.77 (0.50, 1.19)	0.97 (0.60, 1.59)	0.9570

a. Logistic regression adjusted for age, race, drinking status, smoking status, education level, income, total energy intake and physical activity.
b. Odds ratio (95% confidence interval) (all such values).
BMI, body mass index.
c. *P* for trend was analysed by multiple logistic regression analysis.
